# Risk factors, time to onset and recurrence of delirium in a mixed medical-surgical ICU population: A secondary analysis using Cox and CHAID decision tree modeling

**DOI:** 10.17179/excli2021-4381

**Published:** 2022-01-04

**Authors:** Farshid Rahimibashar, Andrew C. Miller, Mahmood Salesi, Motahareh Bagheri, Amir Vahedian-Azimi, Sara Ashtari, Keivan Gohari Moghadam, Amirhossein Sahebkar

**Affiliations:** 1 Department of Anesthesiology and Critical Care, School of Medicine, Hamadan University of Medical Sciences, Hamadan, Iran; 2 Department of Emergency Medicine, Alton Memorial Hospital, Alton, IL, USA; 3 Chemical Injuries Research Center, Systems Biology and Poisonings Institute, Baqiyatallah University of Medical Sciences, Tehran, Iran; 4 Student Research Committee, Hamadan University of Medical Sciences, Hamadan, Iran; 5 Trauma Research Center, Nursing Faculty, Baqiyatallah University of Medical Sciences, Tehran, Iran; 6 Gastroenterology and Liver Diseases Research Center, Research Institute for Gastroenterology and Liver Diseases, Shahid Beheshti University of Medical Sciences, Tehran, Iran; 7 Department of Internal Medicine, Shariati Hospital, Tehran University of Medical Sciences, Tehran, Iran; 8 Biotechnology Research Center, Pharmaceutical Technology Institute, Mashhad University of Medical Sciences, Mashhad 9177948564, Iran; 9 Applied Biomedical Research Center, Mashhad University of Medical Sciences, Mashhad, Iran; 10 Department of Biotechnology, School of Pharmacy, Mashhad University of Medical Sciences, Mashhad, Iran

**Keywords:** delirium, Intensive Care Units, critical care, risk factors, Iran

## Abstract

A retrospective secondary analysis of 4,200 patients was collected from two academic medical centers. Delirium was assessed using the Confusion Assessment Method for the Intensive Care Unit (CAM-ICU) in all patients. Univariate and multivariate Cox models, logistic regression analysis, and Chi-square Automatic Interaction Detector (CHAID) decision tree modeling were used to explore delirium risk factors. Increased delirium risk was associated with exposed only to artificial light (AL) hazard ratio (HR) 1.84 (95 % CI: 1.66-2.044, *P*<0.001), physical restraint application 1.11 (95 % CI: 1.001-1.226, *P*=0.049), and high nursing care requirements (>8 hours per 8-hour shift) 1.18 (95 % CI: 1.048-1.338, *P*=0.007). Delirium incidence was inversely associated with greater family engagement 0.092 (95 % CI: 0.014-0.596, *P*=0.012), low staff burnout and anticipated turnover scores 0.093 (95 % CI: 0.014-0.600, *P*=0.013), non-ICU length-of-stay (LOS)<15 days 0.725 (95 % CI: 0.655-0.804, *P*<0.001), and ICU LOS ≤15 days 0.509 (95 % CI: 0.456-0.567, *P*<0.001). CHAID modeling indicated that AL exposure and age <65 years were associated with a high risk of delirium incidence, whereas SOFA score ≤11, APACHE IV score >15 and natural light (NL) exposure were associated with moderate risk, and female sex was associated with low risk. More rapid time to delirium onset correlated with baseline sleep disturbance (*P*=0.049), high nursing care requirements (*P*=0.019), and prolonged ICU and non-ICU hospital LOS (*P*<0.001). Delirium recurrence correlated with age >65 years (HR 2.198; 95 % CI: 1.101-4.388, *P*=0.026) and high nursing care requirements (HR 1.978, 95 % CI: 1.096-3.569), with CHAID modeling identifying AL exposure (*P*<0.001) and age >65 years (*P*=0.032) as predictive variables. Development of ICU delirium correlated with application of physical restraints, high nursing care requirements, prolonged ICU and non-ICU LOS, exposure exclusively to AL (rather than natural), less family engagement, and greater staff burnout and anticipated turnover scores. ICU delirium occurred more rapidly in patients with baseline sleep disturbance, and recurrence correlated with the presence of delirium on ICU admission, exclusive AL exposure, and high nursing care requirements.

## Introduction

Delirium is a transient fluctuating global disorder of cognition associated with increased morbidity and mortality (Witlox et al., 2010[80]; Girard et al., 2010[22]; Pisani et al., 2009[55], 2010[56]), whose prevalence among intensive care unit (ICU) patients may reach 80 % (Kalabalik et al., 2014[30]), with a daily probability up to 14 % (Schreiber et al., 2014[64]). ICU delirium may be a predictor of increased complications, prolonged ICU (Yamaguchi et al., 2014[81]) and non-ICU hospital length-of-stay (LOS) (Al-Qadheeb et al., 2016[2]), increased hospital costs (Gleason et al., 2015[23]; Vasilevskis et al., 2018[76]), long-term disability (Marcantonio et al., 2005[38]), long-term cognitive impairment (Pandharipande et al., 2013[52]; Sukantarat et al., 2005[70]), and decreased odds of discharge home (Devlin et al., 2012[14]; Shehabi et al., 2010[65]; Tsuruta et al., 2010[72]). Moreover, ICU delirium has been associated with the development of incident neuropsychiatric disorders including depression, anxiety, trauma and stress-related, and neurocognitive disorders (Brown et al., 2020[9]). Therefore, delirium prevention, early diagnosis and treatment are important aspects of caring for the critically-ill patient (Chakraborti et al., 2015[12]; Inouye et al., 2014[27]). The mechanism(s) of delirium remain(s) unclear, and no diagnostic laboratory or imaging test is available (Bashar et al., 2018[6]; Farzanegan et al., 2021[20]). The American Psychiatric Association's Diagnostic and Statistical Manual, 5th edition (DSM-V) defines delirium by: disturbances of attention, cognition, that develops over a short period, differs from baseline, fluctuates not otherwise explained by another neurocognitive disorder, and with evidence suggesting a potential cause in the history, physical examination, or laboratory findings (American Psychiatric Association, 2013[3]). Risk factors are multifactorial and may be divided into patient-related and hospital-related (Kanova et al., 2017[31]). Patient-related factors include: age, gender, underlying disease, baseline cognitive impairment, illness severity (measured as Acute Physiology and Chronic Health Evaluation (APACHE) IV score), and presence of delirium at admission. Hospital-related factors include medications (including sedatives), nursing care, staff burnout and turnover, mechanical ventilation (MV), non-ICU hospital or ICU LOS, isolation, physical restraint application, and artificial vs. natural light exposure (Inouye et al., 1999[26]; Arenson et al., 2013[4]; Vahedian-Azimi et al., 2020[75]). 

This study aims to determine delirium incidence, recurrence rates, and predict the associated risk factors in ICU patients with acute respiratory distress syndrome (ARDS) on MV using univariate and multivariate Cox models, logistic regression analysis, and Chi-square Automatic Interaction Detector (CHAID) decision tree. 

## Material and Methods

### Study design and setting

A prospective cohort study was conducted on 16,000 ICU patients with ARDS on MV from 21 ICUs (10 mixed, five surgical, six medical) at six academic medical centers (Bashar et al., 2018[6]). Herein, a retrospective secondary analysis of 4,200 patients from the mixed medical-surgical ICUs of two academic medical centers is reported (Bashar et al., 2018[6]). To select 4,200 patients from the total prospective cohort; delirium based on CAM-ICU score was categorized into low/high groups. Optimal thresholds were selected by receiver operating curve (ROC) analysis of a database of 16,000 ICU patients. To achieve a sensitivity of 95 % and a specificity of 95 %, for the best ROC characteristics. Thresholds' selection was discussed in a qualitative panel of 31 members including two psychiatrists, three psychologists, five intensivists, three neurologists, three internists, five anesthesiologists, and ten ICU nurses. Consensus agreement was achieved based on the available data (Farzanegan et al., 2021[20]). The study protocol was approved by the Ethics Committee of Hamadan University of Medical Sciences, Hamadan, Iran, with code IR.UMSHA.REC.1400.552. Written informed consent from the patient or designated surrogate was required for participation in the parent study. The manuscript was prepared in accordance with the “Strengthening the Reporting of Observational Studies in Epidemiology (STROBE) Statement” (von Elm et al., 2008[78]). 

### Participants and data collection

All adult patients admitted to mixed medical-surgical ICUs of two academic teaching hospitals from June 1, 2007 to October 31, 2015 were eligible for this study. The inclusion criteria were: (1) age ≥18 years, (2) endotracheal intubated and on MV for ≥48 hours, (3) full-code status, and (4) informed consent obtained from the patient, legal guardian, or healthcare surrogate. Patients were excluded for: (1) if death occurred while on MV, (3) permanent ventilator dependence, (4) tracheostomy placement for long-term weaning, and (5) incomplete data.

### Delirium

Delirium was assessed during each shift (three times a day) by the bedside nurse and researcher (kappa agreement coefficient 0.801-0.902), using the Confusion Assessment Method for the ICU (CAM-ICU) screening tool (Ely et al., 2001[17]). The CAM-ICU allows one to screen for delirium presence (not severity) in critically ill patients, including those on MV (sensitivity 75.5 %, specificity 95.8 %) (Ely et al., 2001[17][18]; Neto et al., 2012[49]). If delirium occurred at any point during the 24-hour period, the day was considered as a day with delirium. Time to onset of ICU delirium was calculated from the patient's physician in the ICU.

### Natural vs. artificial light

The impact of natural light (NL) and artificial light (AL) on delirium was also assessed. Both ICUs had the same geographic layout including 10 beds; five with adjacent windows allowing for NL (circadian pattern), and five positioned 13 m from the nearest window receiving only AL. Patients were categorized according to their original bed locations as NL and AL groups (Vahedian-Azimi et al., 2020[75]). 

### Variables and outcomes

Baseline demographic data was recorded including age, gender, presence of delirium on admission, baseline cognitive impairment (CI) determined by the six-item cognitive impairment test (6-CIT) which assess logical memory (five-items), attention (two items) and orientation (three items) (Hessler et al., 2017[24]). 6-CIT scores range from 0 to 28, with higher scores indicating greater CI. In accordance with prior studies, the threshold of 9/10 was used for delirium screening (O'Sullivan et al., 2018[51]; Lacko et al., 1999[37]; Queally et al., 2010[58]). Scores of 0-7 are considered normal and 8 or more significant.

Comorbidities such as diabetes, hypertension, malignancy, congestive heart failure (CHF), and chronic kidney, liver and pulmonary diseases, which were assessed by the Charlson Comorbidity Index (Deyo et al., 1992[15]; Quan et al., 2005[57]). Activity and mobility as measured by the Perme ICU mobility score (IMS), reflects the patient's mobility status at one particular moment in time (Perme et al., 2014[54]; Wilches Luna et al., 2018[79]). Data was collected by a trained physical therapist. The score is derived from 15 items grouped in 7 categories: mental status, potential mobility barriers, functional strength, bed mobility, transfers, gait (with or without assistive devices), and endurance. The score uses a maximum range of 2 to 4 points for each item, with total scores ranging 0 to 32. In this instrument, a high score indicates high mobility and a reduced need for assistance. In contrast, a low score indicates low mobility and an increased need for assistance (Perme et al., 2014[54]; Wilches Luna et al., 2018[79]).

Hospital-related factors including family engagement (family bedside presence ≥2 hours daily) (Khaleghparast et al., 2015[32]), level of nursing care (determined by requiring >8 hours nursing care in an 8 hour shift staff), burnout and anticipated turnover (measured by the anticipated turnover scale (ATS) questionnaire), (Barlow and Zangaro, 2010[5]; Shoorideh et al., 2015[66]; Kaddourah et al., 2018[29]; Adams et al., 2019[1]), non-ICU hospital and ICU LOS, sedative dose (determined in accordance with published recommendations), (Miller et al., 2015[43]; Nagaraj et al., 2016[47], 2017[46]; Farzanegan et al., 2021[20]), and physical restraint application were collected for each patient. Illness severity was measured by the APACHE IV and Sequential Organ Failure Assessment (SOFA) scores (Knaus et al., 1985[34]; Zimmerman et al., 2006[84]; Vincent et al., 1996[77]). Baseline sleep disturbance was assessed on ICU day one using the Pittsburgh Sleep Quality Index (PSQI) (Buysse et al., 1989[11]). 

### Environmental noise assessment and intervention

Ambient noise level and use of an alarm silence strategy were assessed using the TES 1352A sound level meter (SLM) device (TES Electrical Electronic Corp., Taiwan) with a range of 30-130 decibel (dB). It has a 1.27 cm electret condenser microphone and accuracy of ±1.5 dB (ref 94 dB@1KHz) (Sosa et al., 2018[69]). For the most accurate estimate of what a patient would hear, the sound meter was placed adjacent to the patient's head (or on their pillow if out of the room) for measurements. Measurements were made by the patient's nurse three times daily (10 AM, 5 PM, and 10 PM).

As the World Health Organization (WHO) recommends noise levels in hospitals should be ≤40 dB during the day, and ≤35 dB during the night shift (Buntinx et al., 1992[10]), days were categorized as noisy if ≥1 reading measured >40 dB. Patients were then grouped according to environmental noise into more noise (≥50 % days with measurements ≥40 dB) and less noise groups (<50 % days with measurements ≥40 dB). 

At our center, we have a noise control policy to minimize sound by utilizing an alarm silence strategy which was accentuated during the study. The alarm load is minimized by setting alarms based on the patient's condition and planned reduction of unnecessary alarms. For example, alarms are silenced proactively when performing bedside procedures such as endotracheal tube suctioning, phlebotomy, and when handling invasive lines. Alarms are then reset upon task conclusion. Additionally, ambient noise is reduced by muting personal phones, limiting unnecessary staff conversation in patient care and common areas. After the alarm silence strategy, noise intensity was measured again in the same locations using a sound meter in dB. If the level of noise was reduced<40 dB, this item would be considered as positive for patients. 

### Statistical analysis

All analyses were performed using IBM® SPSS® 23.0 (IBM Corp., Armonk, NY) (Kline, 1998[33]) and GraphPad Prism 5© (GraphPad Software Inc., La Jolla, CA). Descriptive statistics were calculated for all variables. Categorical variables were expressed as counts (percentage) and continuous variables as mean ± standard deviation (SD). Patients were stratified by the occurrence or absence of delirium during the ICU LOS, and demographic and clinical characteristics were assessed using t-test with continuous variable and Chi-Square, or Fisher's Exact test (as appropriate) with categorical variables.

Univariate and multivariate Cox models were separately used to assess predictors of delirium incidences and the time to delirium onset. In the Cox model, the time to delirium onset was the main predictor. In the multivariate analyses, the significant variables in a backward selection modeling (considering Pentry =0.05 and Premoval =0.10) were reported as hazard ratio (HR) with 95 % CI, also; a multivariate linear regression was used to predict the occurrence of delirium. In addition, multivariate logistic regression was used to identify those factors exerting a statistically significant effect on the incidence of delirium recurrence by using backward method and the significant variables were reported as odds ratio with 95 % CI.

Chi-square Automatic Interaction Detector (CHAID) decision tree analysis is a data mining technique which can demonstrate the relationship between split variables and related factors in homogeneous population subgroups (Song and Lu, 2015[68]). Moreover, CHAID enables one to deal with whole variables, partition consecutive data effectively, and makes decision trees by using a forward stopping or pruning rule (Gandomi et al., 2013[21]; Miller et al., 2014[42]). For CHAID decision tree analysis in this study, all parameters collected for delirium incidence and recurrence were used. The minimum parent and child nodes were determined as 100 and 50, respectively. “Nodes” are midpoints or terminal points after bifurcation according to each factor. The parent nodes are the nodes before bifurcation, and the child nodes are ones after bifurcation. Based on the result, a group of patients was divided into one of the terminal nodes (risk groups) with calculated predictive probability. Significance was determined as an alpha of 0.05.

## Results

### Study population

A total of 4,200 subjects were included in the analysis. Demographic and clinical characteristics are presented in Table 1(Tab. 1). The mean participant age was 67.25±11.5 years, with more than half with age >65 years (51.2 %) with a female preponderance (58.1 %). No significant differences were noted in these variables between those who did and did not develop delirium. Delirium was identified in 1,540 (36.7 %) of patients during the ICU stay. The mean time to delirium recognition was 7.55±1.88 days. The majority of patients did not have documented comorbidities (n=3,289, 78.3 %, Table 1(Tab. 1)), and comorbidities did not differ between groups (*P*=0.102). However, of those 911 with documented comorbidities based on Charls on comorbidity index which includes 217 (23.8 %) CHF, 183 (20.1 %) malignancy, 145 (15.9 %) chronic kidney diseases, 129 (14.1 %) chronic liver diseases, 121 (13.2 %) diabetes mellitus, 84 (9.2 %) chronic pulmonary diseases, 16 (1.7 %) hypertension and 16 (1.7 %) arthritis. 

Non-ICU hospital LOS (*P*=0.584) and ICU LOS (*P*=0.552) was similar between groups. Illness severity as measured by the APACHE IV and SOFA score was similar between groups (*P*=0.357) and (*P*=0.305), respectively.

Patients with and without delirium differed significantly in terms of cognitive impairment at the time of admission. Baseline cognitive impairment significantly was higher in patients with delirium (33.7 % vs. 7.4 %, *P*<0.001). Additionally, a greater portion of delirium patients (62.7 %) were observed in the AL group (*P*<0.001). Other characteristics did not differ significantly between groups (*P*>0.05) (Table 1(Tab. 1)). 

### Development of delirium

**Figure 1:**
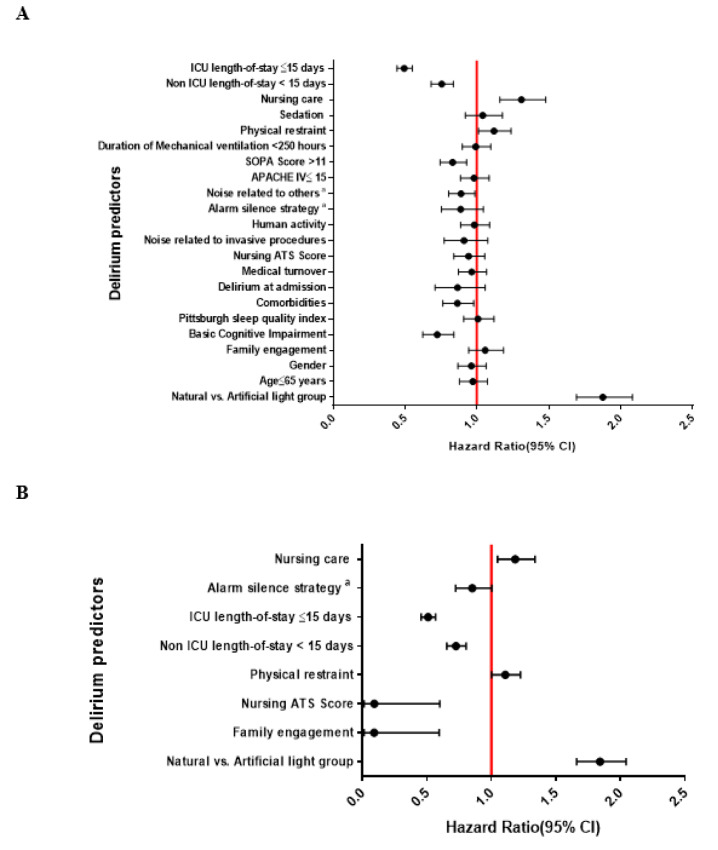
Univariate (A) and multivariate (B) Cox regression analyses to identify factors predictive of developing ICU delirium. Abbreviations: ATS means anticipated turnover scale; APACHE IV means Acute Physiology and Chronic Health Evaluation IV; MV means mechanical ventilator; LOS means length of stay,^ a^ Noise related to the nursing stations, staff conversation in patients' bedside and medical devices.

The results of the univariate and multivariate Cox regression analyses which predict the risk of developing delirium are presented in Figures 1(Fig. 1) and Table 2(Tab. 2). On multivariate analysis, patients were at increased risk of developing delirium if: (1) were categorized in the AL cohort (hazard ratio (HR): 1.84, 95 % CI: 1.66-2.044, *P*<0.001), (2) had applied physical restraints (HR: 1.11, 95 % CI: 1.001-1.226, *P*=0.049), and (3) required more nursing care (>8 hours per 8 hour shift; HR: 1.18, 95 % CI: 1.048-1.338, *P*=0.007). Notably, the application of physical restraints was associated with a 10 % increased risk of delirium, whereas greater nursing care requirements were associated with an 18 % increased risk. 

Conversely, lower delirium rates were associated with: (1) greater family engagement (HR: 0.092, 95 % CI: 0.014-0.596, *P*=0.012), (2) low staff burnout and anticipated turnover (ATS ≤3.5; HR: 0.093, 95 % CI: 0.014-0.600, *P*=0.013), (3) non-ICU hospital LOS <15 days (HR: 0.725, 95 % CI: 0.655-0.804, *P*<0.001), and (4) ICU LOS ≤15 days (HR: 0.509,95 % CI: 0.456-0.567, *P*<0.001). 

**Figure 2:**
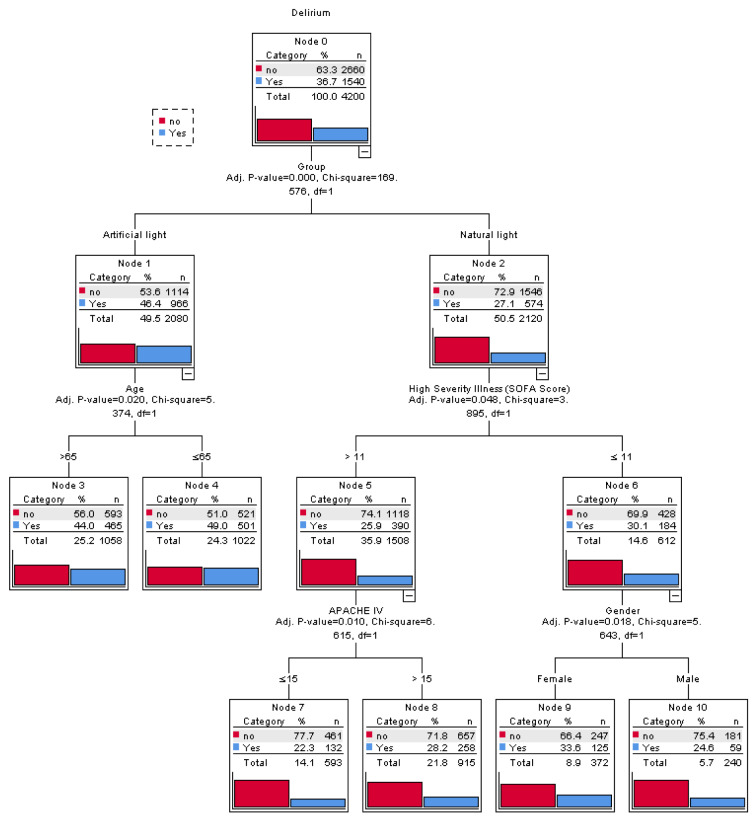
A CHAID decision classification tree analysis to predict delirium among participants

Figure 2(Fig. 2) depicts the CHAID decision tree analysis for predicting delirium incidence among all participants (n=4,200). Five variables were used for grouping in the decision tree model: type of light exposure, age, SOFA score, APACHE IV score, and gender. The model includes the total of 11 nodes, three intermediate nodes and six terminal nodes. Each node contains three statistical values; category, percentage ( %) and the number (n) of patients in this particular category. First, subjects were compared according to type of light exposure. In the AL and NL groups, age and SOFA score were assessed, respectively. If the SOFA score was >11, then the APACHE IV score was checked. If the SOFA score was ≤11, then patient sex was assessed. Subjects were then stratified according to the risk of delirium incidence into low (<20 %), moderate (20-30 %), high (30-40 %), and very high (>40 %) risk groups. The findings suggest that AL and age <65 years conveyed a high risk of delirium incidence, whereas SOFA score ≤11 and female sex were associated with low risk, and APACHE IV score (>15) and NL were associated with moderate risk. 

### Time to delirium onset

Multivariate linear regression analysis was conducted to identify those variables predictor of time to delirium onset. As shown in Table 3(Tab. 3), sleep disturbance, high nursing care requirements (>8 h per 8 h shift) and non-ICU hospital LOS>15 days correlated with shorter time to delirium onset. Notably, delirium occurred 1.12 days earlier in patients with baseline sleep disturbance (*P*=0.049). In terms of nursing care hours required per 8 hours shift, delirium occurred 1.55 days later in patients requiring mild (<4 hours) to moderate (4-8 hours) nursing care than in those requiring a high level (>8 hours) of nursing care (*P*=0.019). Lastly, delirium occurred 10.48 days earlier in patients with a non-ICU hospital LOS>15 days prior to ICU admission (*P*<0.001).

### Delirium recurrence

Multivariate logistic regression was used to identify those factors exerting a statistically significant effect on the incidence of delirium recurrence by using backward method and the significant variables were reported as odds ratio (OR) in 320 patients (7.6 %) with delirium at the time of admission. The results, as shown in Table 4(Tab. 4), indicate that AL (HR: 3.239, 95 % CI: 1.881-5.577, *P*<0.001), high nursing care (>8 hours per shift; HR1.978, 95 % CI: 1.096-3.569, *P*=0.024), and age>65 years (HR: 2.198, 95 % CI: 1.101-4.388, *P*=0.026) were associated with increased rates of delirium recurrence. 

**Figure 3:**
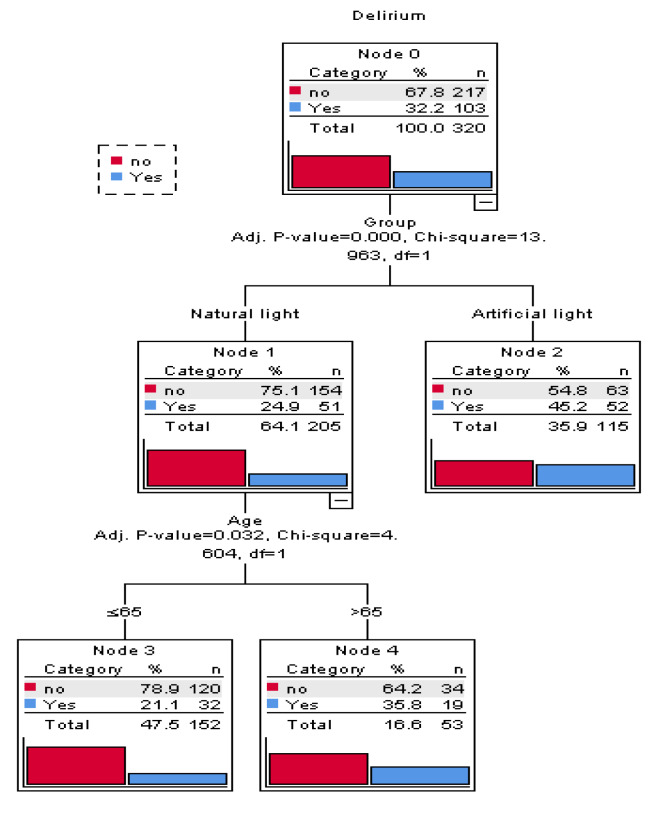
A CHAID decision classification tree analysis to predict delirium recurrence in patients with delirium at the admission time

Figure 3(Fig. 3) depicts the CHAID decision tree analysis for predicting delirium recurrence in patients with delirium present on ICU admission (n=320). This decision tree has a depth of two levels from the root node, with one intermediate node, and three terminal nodes. Each node contains three statistical values, category, percentage ( %) and the number (n) of patients in this particular category. As shown in Figure 3(Fig. 3), the main variables associated with delirium recurrence were AL exposure (*P*<0.001) and age >65 years (*P*=0.032).

See also the Supplementary data.

## Discussion

Delirium is a common and serious clinical syndrome characterized by fluctuating cognitive dysfunction that affects 20 % to 80 % of ICU patients (Martinez et al., 2012[39]; Ryan et al., 2013[62]). The risk of delirium relies on the interaction between predisposing and precipitating risk factors (Ely et al., 2001[18]; Kanova et al., 2017[31]). It is associated with increased short- and long-term morbidity and mortality (Witlox et al., 2010[80]; Girard et al., 2010[22]; Pisani et al., 2009[55], 2010[56]; Kalabalik et al., 2014[30]; Yamaguchi et al., 2014[81]; Al-Qadheeb et al., 2016[2]; Gleason et al., 2015[23]; Marcantonio et al., 2005[38]; Pandharipande et al., 2013[52]; Sukantarat et al., 2005[70]; Devlin et al., 2012[14]; Shehabi et al., 2010[65]; Tsuruta et al., 2010[72]; Brown et al., 2020[9]). Thus, a thorough understanding of mitigating and contributing factors is necessary to develop an accurate delirium prediction model for critically ill patients.

The incidence of delirium in this study (36.7 %) was consistent with that of some published studies (Salluh et al., 2015[63]; Jayaswal et al., 2019[28]), but lower than some other cohorts (Girard et al., 2010[22]; Shehabi et al., 2010[65]). The median time to ICU delirium onset was similar to other published studies (Heymann et al., 2007[25]; Tilouche et al., 2018[71]). Moreover, the seven variables identified on Cox regression analysis were similar to other published reports (Vahedian Azimi et al., 2015[74]; Kobayashi et al., 2013[35]; Jayaswal et al., 2019[28]) including light category (artificial vs. natural), low level of family engagement (<2 hours at bedside per day), high nurse burnout and anticipated turnover (ATS>35), application of physical restraints, high nursing care requirements (>8 hours in 8 hours shift), ICU LOS > 15 days, and hospital LOS >15 days. The most predictive variables of developing delirium on CHAID decision tree modeling were AL group and age >65 years (high risk), APACHE IV score >15 (moderate risk), SOFA score ≤11 and female sex (low risk). As it pertains to light exposure, loss of NL exposure is associated with circadian rhythm disturbances that may affect delirium incidence and outcomes in the critically ill patients (Boyko et al., 2017[8]; Oldham et al., 2016[50]; Vahedian-Azimi et al., 2020[75]; Kohn et al., 2013[36]). The connection of NL vs. AL light exposure and delirium incidence has been variably reported (Smonig et al., 2019[67]; Estrup et al., 2018[19]; Zaal et al., 2013[83]; Vahedian-Azimi et al., 2020[75]). This discrepancy may be related to differences in delirium definition, screening method, NL category criteria, and sample size (Vahedian-Azimi et al., 2020[75]). 

Beyond grouping by light exposure type, CHAID analysis further identified the female gender, SOFA >11, and APACHE IV >15 as risk factors in the second and third layer of the decision tree model. These factors were likely not detected in Cox regression analysis because of higher proportion of females in participants and the similar median score of APACHE IV and SOFA in two groups. In fact, one advantage of the CHAID decision tree is that it can divide the population into subgroups with different characteristics and estimate the prevalence in each subgroup. While, regression analysis examines risk factors throughout the whole population and treats different factors equally (Ye et al., 2016[82]). However, we believed both models were clinically reasonable.

According to the Cox regression analysis, high nursing care and use of the physical restraint predisposed patients to 18 % and 10 % greater risk of delirium, respectively, and it is consistent with the other studies in this field (McPherson et al., 2013[40]; Mehta et al., 2015[41]). Physical restraints are often used for critically ill patients to ensure patient safety, and prevent the removal of medical equipment (e.g., tracheal tubes) (Unoki et al., 2019[73]). However, the use of physical restraints in different countries varies considerably. For example, the use of physical restraints in European general ICU populations ranges from 10 % to 50 %, 76 % in Canada, and up to 87 % in American surgical ICUs (Perez et al., 2019[53]; Benbenbishty et al., 2010[7]; De Jonghe et al., 2013[13]). According to one meta-analysis, the prevalence of physical restraint use in Iranian medical-surgical ICUs was 47.6 %, in keeping with the findings of this analysis (Moradimajd et al., 2015[44]). Similarly, physical restraint applications have previously been identified as an independent risk factor for development of ICU delirium (Mori et al., 2016[45]; Perez et al., 2019[53]). As restraint use increased two- and three-fold, observed incidence of ICU delirium increased 2.38- and 3.62-fold, respectively.

Additionally, the presence of family at bedside for >2 hours per day (reported as family engagement) was identified as a potential mitigating factor for ICU delirium, similar to other published reports (Rosenbloom-Brunton et al., 2010[61]; Eghbali-Babadi et al., 2017[16]). This raises questions about the role that family may play in the care of a critically-ill loved one and presents an opportunity for inquiry as ICU visitation policies have been restricted in many cases during the current COVID-19 pandemic. Current evidence suggests that this may potentially be accomplished in the confines of traditional visiting hours, rather than more flexible visitation policies that may contribute to staff burnout (Rosa et al., 2019[60]; Nassar Junior et al., 2018[48]). 

Healthcare provider turnover is an important indicator for care quality and is widely used as a measure for health-care system analysis. Burnout and provider turnover may disrupt patient care quality and continuity (Reddy et al., 2015[59]; Kaddourah et al., 2018[29]; Shoorideh et al., 2015[66]; Adams et al., 2019[1]). However, whether provider burnout is linked to patient development of ICU delirium remains unclear. In the current study, provider burnout and intent for job turnover was assessed as regards to its correlation to development of ICU delirium. This study found that delirium risk was higher in patients whose providers had higher rates of burnout and anticipated turnover as measured by ATS scores (HR 0.093, 95 % CI: 0.014-0.600, *P*=0.013).

To identify factors predictive of delirium recurrence amongst those patients with delirium at ICU admission, backward logistic regression analysis and CHAID decision tree modeling identified exclusive AL light exposure and age >65 years as major risk factors in the present study. Similar to prior studies, hospitalization in a room without NL exposure was associated with a 3.24-fold increase in delirium recurrence (Vahedian-Azimi et al., 2020[75]), whereas age >65 years increased delirium recurrence by 2.19-fold (Tilouche et al., 2018[71]). This may not be entirely surprising, as the elderly may be more susceptible to the effects of metabolic disturbances, hypoxemia, and other stresses imposed by the critically ill state (Tilouche et al., 2018[71]). It remains unclear whether the high levels of nursing requirements associated with increased delirium recurrence are merely a reflection of patients with more severe illness or delirium, or it correlates with an as-yet unmeasured risk factor. 

This report details the largest study of its type on ICU delirium. More than twenty related factors were analyzed using two different prediction model methods. Nevertheless, this study is not without limitations. First, our prediction model method requires knowledge of the patient's medical history. In some cases, this may be limited by recall bias, or non-availability of information. Second, it's related to the inherent limitations of an observational study design.

## Conclusion

Development of ICU delirium correlated with application of physical restraints, high nursing care requirements, prolonged ICU and non-ICU hospital length-of-stay, exposure exclusively to artificial (rather than natural) lighting, less family engagement, and greater staff burnout and anticipated turnover scores. ICU delirium occurred more rapidly in patients with baseline sleep disturbance, and recurrence correlated with presence of delirium on ICU admission, exclusive artificial light exposure, and high nursing care requirements. Many of these factors are suitable for further studies and interventions such as natural light exposure, and minimizing physical restraint application and, most notably, the potential impacts of provider burnout and intent to turnover on patient's development of ICU delirium. 

## Notes

Mahmood Salesi and Amirhossein Sahebkar (Biotechnology Research Center, Pharmaceutical Technology Institute, Mashhad University of Medical Sciences, Mashhad 9177948564, Iran; Tel: +985138002299, Fax: +985138002287, E-mail: sahebkara@mums.ac.ir, amir_saheb2000@yahoo.com) contributed equally as corresponding author.

## Declaration

### Conflicts of interest

The authors declare no conflict of interest.

### Ethical approval

This study was conducted in accordance with the guidelines of the Declaration of Hel-sinki and was reviewed and approved by the Ethics Committees of Hamadan University of Medical Sciences (IR.UMSHA.REC.1400.552). 

### Funding 

This research did not receive any specific grant from funding agencies in the public, commercial, or not-for-profit sectors.

### Availability of data and material

The datasets used and/or analyzed during the current study are available from the corresponding author on reasonable request.

### Acknowledgments 

The study was supported by vice chancellor for Research and Technology, Hamadan University of Medical Sciences. Moreover, thanks to guidance and advice from the "Clinical Research Development Unit of Baqiyatallah Hospital".

## Supplementary Material

Supplementary data

## Figures and Tables

**Table 1:**
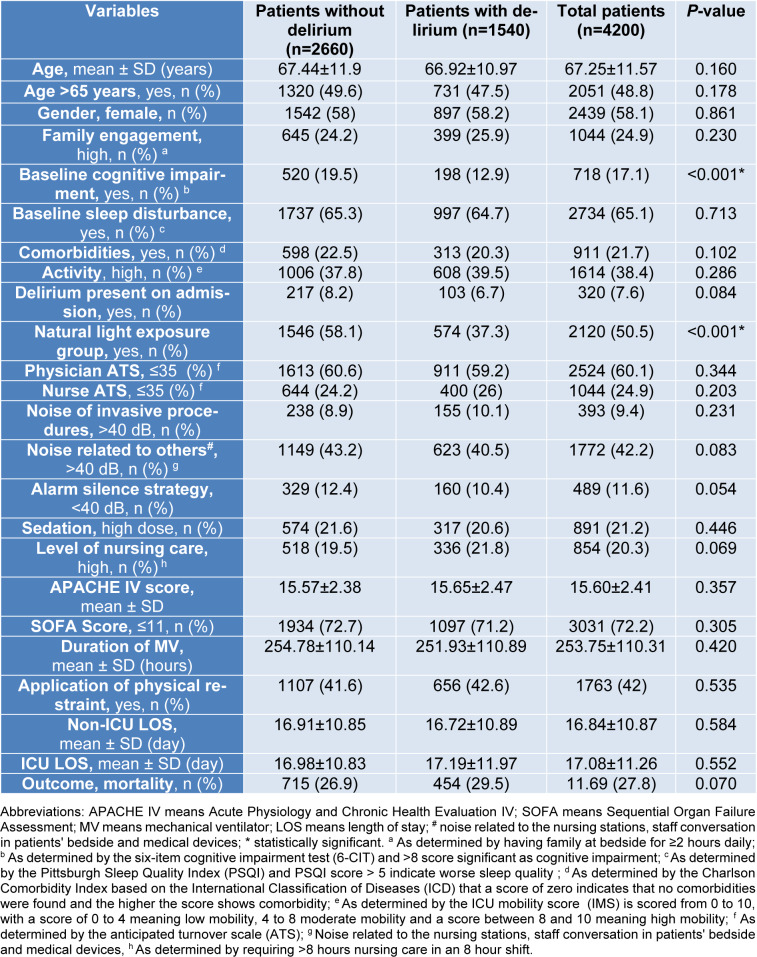
Demographic and clinical characteristics of the participants according to with and without delirium

**Table 2:**
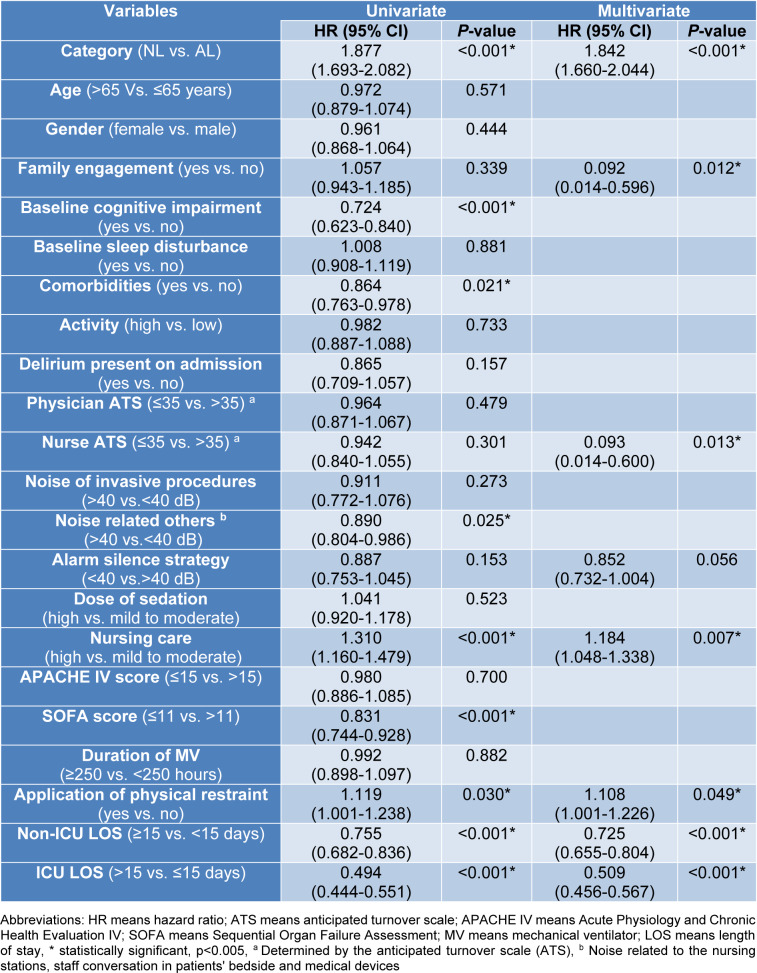
Univariate and multivariate Cox regression analysis of influencing factors to predict delirium incidence

**Table 3:**
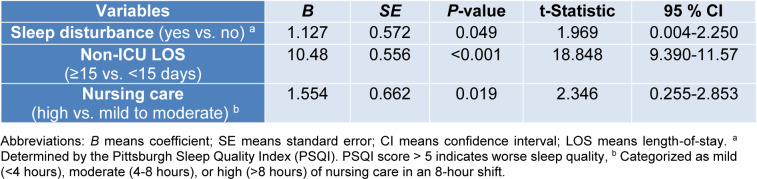
Linear regression analysis of influencing factors to predict time incidence of delirium

**Table 4:**
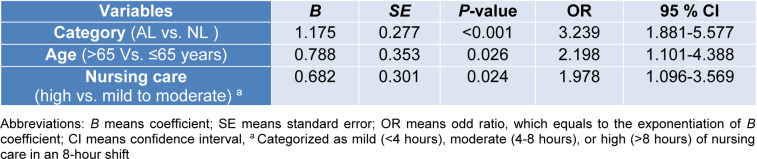
Backward logistic regression analysis of influencing factors to predict delirium recurrence in patients with delirium at the admission time
